# Predictive QSAR model confirms flavonoids in Chinese medicine can activate voltage-gated calcium (CaV) channel in osteogenesis

**DOI:** 10.1186/s13020-020-00313-1

**Published:** 2020-03-31

**Authors:** Ki Chan, Henry Chi Ming Leung, James Kit-Hon Tsoi

**Affiliations:** 1 Dental Materials Science, Division of Applied Oral Sciences and Community Dental Care, Faculty of Dentistry, The University of Hong Kong, Pokfulam, Hong Kong SAR PRC; 2 Department of Computer Science, Faculty of Engineering, University of Hong Kong, Pokfulam, Hong Kong SAR PRC

**Keywords:** QSAR, Flavonoids, Voltage-gated calcium channels, Computer modelling

## Abstract

**Background:**

Flavonoids in Chinese Medicine have been proven in animal studies that could aid in osteogenesis and bone formation. However, there is no consented mechanism for how these phytochemicals action on the bone-forming osteoblasts, and henceforth the prediction model of chemical screening for this specific biochemical function has not been established. The purpose of this study was to develop a novel selection and effective approach of flavonoids on the prediction of bone-forming ability via osteoblastic voltage-gated calcium (CaV) activation and inhibition using molecular modelling technique.

**Method:**

Quantitative structure–activity relationship (QSAR) in supervised maching-learning approach is applied in this study to predict the behavioral manifestations of flavonoids in the CaV channels, and developing statistical correlation between the biochemical features and the behavioral manifestations of 24 compounds (Training set: Kaempferol, Taxifolin, Daidzein, Morin, Scutellarein, Quercetin, Apigenin, Myricetin, Tamarixetin, Rutin, Genistein, 5,7,2′-Trihydroxyflavone, Baicalein, Luteolin, Galangin, Chrysin, Isorhamnetin, Naringin, 3-Methyl galangin, Resokaempferol; test set: 5-Hydroxyflavone, 3,6,4′-Trihydroxyflavone, 3,4′-Dihydroxyflavone and Naringenin). Based on statistical algorithm, QSAR provides a reasonable basis for establishing a predictive correlation model by a variety of molecular descriptors that are able to identify as well as analyse the biochemical features of flavonoids that engaged in activating or inhibiting the CaV channels for osteoblasts.

**Results:**

The model has shown these flavonoids have high activating effects on CaV channel for osteogenesis. In addition, scutellarein was ranked the highest among the screened flavonoids, and other lower ranked compounds, such as daidzein, quercetin, genistein and naringin, have shown the same descending order as previous animal studies.

**Conclusion:**

This predictive modelling study has confirmed and validated the biochemical activity of the flavonoids in the osteoblastic CaV activation.

## Background

Flavonoids are polyphenol compounds that are categorized according to their chemical structures into distinct groups, namely flavonols, flavones, flavanones, flavan-3-ols, and isoflavones. In fact, these flavonoids are widely presented in various agricultural food, natural products and Chinese Medicine, and they can exert various health promoting effects in the human body based on their chemical structures [1]. In particular, flavonols are a class of flavonoids, and some compounds, e.g. kaempferol, quercetin, quercetrin, rutin and myricetin are commonly found in *Hippophae rhamnoides*, *Hypericum perforatum*, and *Cacumen platycladi* [2]. In addition, from *Fructus viticis* and *Perilla frutescens*, various flavones such as apigenin, isovitexin, luteolin and vitexin could be obtained [3, 4]. Favones is another class of flavonoids that could be found easily in *Radix scutellariae* and *Cuscuta chinensis.* For example, naringenin can reduce cholesterol levels [5], hesperidin can reduce inflammation via its suppression pathways of lipopolysaccharide (LPS)-elicited and infection-induced Tumor necrosis factor alpha (TNF-α) production [6], and naringin can be used in bone graft material to induce osteogenesis [7]. Flavan-3-ols include the catechins and the catechin gallates. The major compounds are catechin, epicatechin, catechin gallate and epicatechin gallate which are the active components of green tea leaves (*Camellia sinensis*) and have antimutagenic, antitumour, anti-inflammatory and free-radical scavenging activities [8]. Isoflavones has its main sources in soy cheese, soy flour, soybean and tofu, etc. Daidzein and genistein are among several known isoflavones [9].

Some flavonoids, such as daidzein [10], quercetin [11], genistein [12] and naringin [7], have been proven in animal studies that could aid in osteogenesis and bone formation. However, there is no consented mechanism for how these chemicals action on the bone-forming osteoblasts. Some evidences have shown bone resorption [13], cell proliferation [14] and cell signal transport [15] were related to activation of osteoblastic calcium channels. In particular, L-type calcium channels (e.g. CaV1.2) could mediate the change of Ca^2+^ inside the osteoblasts by some regulatory agents such as parathyroid hormone (PTH) [16] and vitamin D [17]. However, study also showed the inhibition of the channels might also promote osteoblast differentiation [18]. On the other hand, Saponara et al. [19] has recently shown in the whole-cell patch-clamp experiments that 24 flavonoids are either activators or deactivators of CaV1.2 channel current measured in artery myocytes of rat tail. Thus, it seems to that the actual physiological mechanisms are unclear [20].

To establish the correlation for the flavonoids with known biochemical activity of being a blocker or activators of Ca^2+^ channels, quantitative structure–activity relationship (QSAR) modeling might be useful to identify and screen the flavonoids, since QSAR could predict biochemical activities for new or untested flavonoids of the same class via selected molecular characteristics (descriptors) that has correlation with the biochemical activity of activating or blocking CaV channels. In fact, QSAR has been used in the fields of medicine, biochemistry, molecular biology and biomaterial science for more than three decades. QSAR is particular useful in screening and predicting the biochemical interaction between, for example, enzyme and complex phytocompounds [21]. Recently, predictive QSAR can be operated in either small, focused and good biochemical data (so-called supervised machine learning approach) that can easily map the biological responses with input feature parameters [22], or utilizing on big enough data for “black box” (so-called deep learning or descriptor-free approach) [23] that can assist in drug design even the chemicals are not existed [24].

Thus, to consider the specific effects of flavonoids on CaV channel current kinetics, a supervised machine learning QSAR model can be built based on chemical structures together with suitable biochemical data (molecular descriptors), such as pKa and patch-clamp experiment data, of the flavonoids to provide a better understanding of the structure–activity relationship between the compounds and CaV. These descriptors have been selectively chosen for the flavonoids since there have been increasing amount of molecular descriptors represented by quantum-chemical and various classical parameters that were designed and tested as potential variables for QSAR modeling. Quantum-chemical parameters represent a special class of molecular properties. They can be obtained from sophisticated ab initio calculations or by means of relatively inexpensive semi-empirical methods, but in the case of flavonoids, such calculations require more time and effort than those for one, two or three-dimensional classical parameters which can be computed from molecular structures of flavonoids within a few minutes. However, in contrast to most classical descriptors, quantum-chemical parameters are capable of expressing all the electronic and geometric properties of the flavonoids being analyzed as well as their interactions [25]. Therefore, the interpretation of quantum-chemical descriptors can provide much deeper insights into the nature of flavonoids’ biochemical and physicochemical mechanisms than that of classical descriptors. The advantages of descriptors calculated by means of quantum-chemical approaches that account for specific and non-specific solvation effects are of prime importance.

This paper focuses on QSAR studies by applying the quantum descriptors based on the current literatures’ biochemical data in predicting the effects of flavonoids’ biochemical activities on the osteoblast’s CaV channel current in order to demonstrate its important biochemical and physicochemical properties on either activating or deactivating the CaV channel for osteogenesis. As such, we anticipate a novel selection and effective approach of flavonoids for CaV activation and inhibition could be developed.

## Materials and methods

QSAR equation modeling attempted to calculate the mathematical correlations between the tested compounds’ chemical attributes and its biochemical response. Such attempt aimed to establish statistical formalism that was indicated as biochemical response of flavonoid = *f* (flavonoids’ biochemical attributes). The flavonoids’ biochemical attributes and property were derived from the flavonoid’s chemical structure and property. Hence, the QSAR equation was expressed as:

$${\text{Biochemical}}\;{\text{response}} = f({\text{flavonoid's}}\;{\text{chemical}}\;{\text{structure}}\;{\text{and}}\;{\text{property}}).$$

To be stated in a simple way, the QSAR equation could be statistically expressed as:

$$Y = \beta_{0} + \beta_{1} X_{1} + \beta_{2} X_{2} + \beta_{3} X_{3} + \cdots + \beta_{n} X_{n} ,$$

where $\beta_{0}$ was a constant; $\beta_{1, } \beta_{2, } \beta_{3, } \ldots \beta_{n}$ were the inputs of descriptors; $X_{1, } X_{2, } X_{3, } \ldots X_{n}$ were different flavonoids’ structural features; and *Y* was biochemical response.

### Steps of QSAR modeling

The four basic steps of QSAR study included (i) data preparation, (ii) data processing, (iii) data prediction and validation, and (iv) data interpretation. The first step was allowed to arrange the data in a convenient and usable form. Since biochemical responses of the flavonoids on CaV channel were considered as the dependent variable, the input data were the flavonoids’ rate of activation and inhibition that were retrieved from Saponara et al. [19]. The predictor variables (i.e. molecular descriptors) could be obtained from chemical structure and property of the flavonoids. After the determination and computation of descriptors, a QSAR table was formed that was a two-dimensional (2D) array of numbers with the columns representing descriptors and response and compounds were depicted in successive rows. As QSAR was basically a statistical approach, the number of observations was higher than the number of descriptors used in the final models for achieving sufficient modeling reliability and robustness. By considering the presence of intercorrelated and redundant data, a pretreatment procedure was also used in the data-processing step.

In each step of the QSAR model development, several statistical operations were involved right from the generation of descriptors which were encoding of information to the pretreatment of data, classification of the data set, development of model, validation and reliability check of the model. Although the partial least squares (PLS) and multiple linear regression (MLR) were common statistical tools to develop QSAR models with genetic algorithm (GA) serving as variable selection methods, these techniques might be inappropriate if $X_{ij}$ is highly correlated or high dimensional, especially in comparison to sample size that might cause variable selection procedures to be unstable.

$$Y_{i} = \beta_{0} + \sum \beta_{j} X_{ij} + \varepsilon_{i} .$$

In these cases, it could find the way to reduce the amount of covariate information because the main focus was on future prediction.

Instead, $\xi_{j}$ was the principle components (PCs) of $X_{\text{i}}$:

$$X_{\text{i}} = \mathop \sum \limits_{j = 1}^{p} \alpha_{ij} \xi_{j} .$$

Then, the principle components regression (PCR) model was developed

$$Y_{i} = \beta_{0}^{'} + \mathop \sum \limits_{j = 1}^{{p^{\prime}}} \beta_{j}^{'} \alpha_{ij} + \varepsilon_{i} ,$$

for some $$p^{\prime} < p.$$

**Fig. 1:**
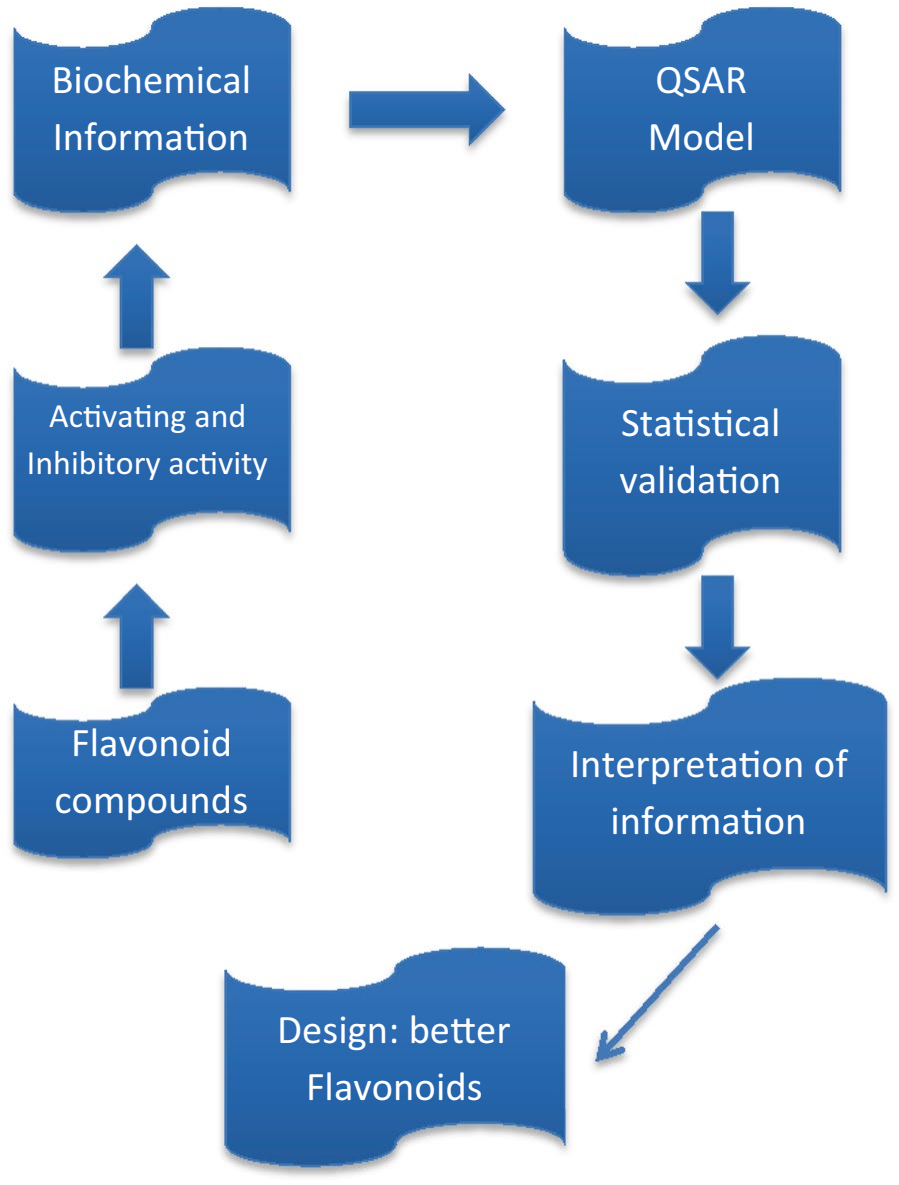
Flowchart of the QSAR formalism

PCR had the advantages that α_ij_ were uncorrelated to strengthen the stability of estimates, and established stable variable selection through dimension reduction. By choosing $p^{\prime}$, enough PCs could be used to do variable selection, capture higher percentage of variation, and maximize adjusted r^2^. The basic workflow of QSAR analysis along with the principal component regression (PCR) was depicted in Fig. 1. On successful runs of Principal Component Regression (PCR) by the QSAR Module of the VLifeMDS 4.3 software (VLife Technologies, Pune, India), the QSAR equations were generated to statistically analyze and determine the model.

### Computation of molecular descriptors

**Fig. 2:**
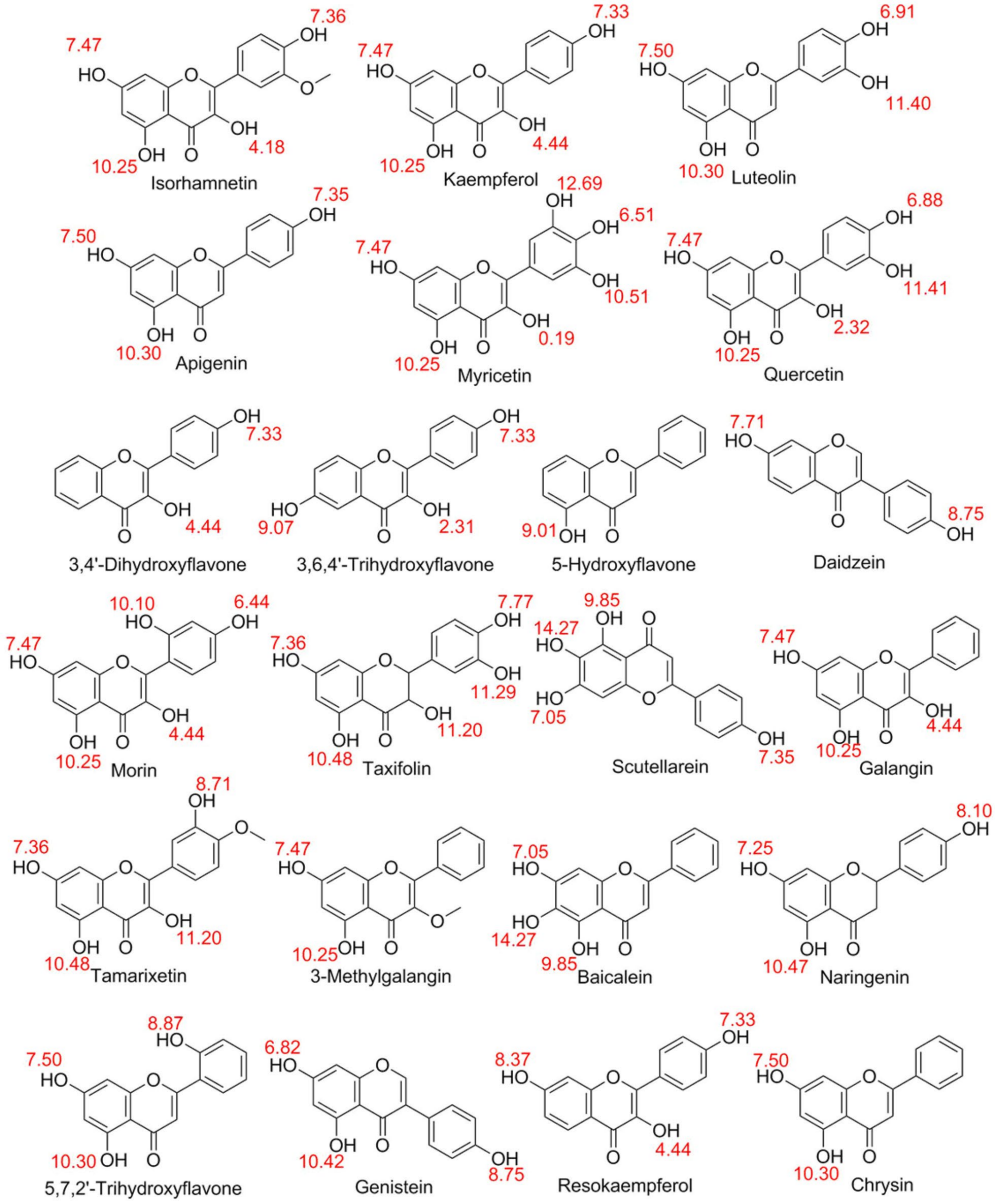
Predicted pKa values (in red colour) for the flavonoids used in this study (Source: PubChem)

First, the QSAR software was applied to align the 3D structural data of the flavonoids as shown in Fig. 2 in order to investigate the variation of molecular shape of each molecule. There had been 20 flavonoids to be included in the training set for deriving QSAR model whereas the chemical structures classified by subclass of flavonoids are shown in Table 1 and the acid dissociation constant (pKa) (i.e. − log10 Ka) values of the hydroxyl groups are shown in Fig. 2. Moreover, the data of the rate of activation and inhibition from Saponara et al. [19] are supplemented as the dataset in which the current evoked at 0 mV from a V_h_ of − 50 mV activated and inhibited with τ of activation ranging between 2.2 and 3.1 ms, and τ of inactivation between 92.0 and 127.9 ms are shown in Table 2. Molecular descriptors, which characterized specific information about a flavonoid, were the numerical value affiliated with the biochemical response for correlation of chemical structures with various biochemical properties. In other words, the modeled response was represented as a function of quantitative values of structural features or properties that were termed as descriptors for the QSAR model. Ab initio derived electronic properties in combination with topological quantum-chemical descriptors (i.e. “k2alpha”, “Id”, “IdwAverage”, “Most+vePotential”, “MomInertiaY” and “DeltaEpsilonC”) were used to help to describe the electronic environment of the flavonoids and locate molecular regions responsible for given bioactivity of flavonoids on the CaV channel [26].

**Table 1:** Chemical structure of flavonoids classified by subclass of flavonoids

Subclass	Name	Substitution		
		OH	OCH_3_	Others
Flavonol	Myricetin	3, 5, 7, 3′, 4′, 5′		
Flavonol	Quercetin	3, 5, 7, 3′, 4′		
Flavonol	Genistein	5, 7, 4′		
Flavonol	Isorhamnetin	3, 5, 7, 4′	3′	
Flavone	Luteolin	5, 7, 3′, 4′		
Flavone	Apigenin	5, 7, 4′		
Flavone	Chrysin	5, 7		
Flavonol	Kaempferol	3, 5, 7, 4′		
Flavonol	Tamarixetin	3, 5, 7, 3′	4′	
Flavonol	Rutin	5, 7, 3′, 4′		*O*-Rutinose
Flavanone	(±)-Taxifolin	3, 5, 7, 3′, 4′		
Flavonol	3,6,4′-Trihydroxyflavone	3, 6, 4′		
Flavone	5,7,2′-Trihydroxyflavone	5, 7, 2′		
Flavone	Scutellarein	5, 6, 7, 4′		
Flavanone	Naringin	5, 4′		*O*-β-Neo-hesperidose
Flavone	5-Hydroxyflavone	5		
Flavonol	3,4′-Dihydroxyflavone	3, 4′		
Isoflavone	Daidzein	7, 4′		
Flavonol	Morin	3, 5, 7, 2′, 4′		
Flavanone	(±)-Naringenin	5, 7, 4′		
Flavonol	Resokaempferol	3, 7, 4′		
Flavone	Baicalein	5, 6, 7		
Flavonol	3-Methyl galangin	5, 7	3	
Flavonol	Galangin	3, 5, 7

**Table 2:** Rate of activation and inactivation of flavonoids on CaV channel under control conditions

Flavonoids	τ_act_ (ms)		τ_inact_ (ms)		*n*
	Control	Drug	Control	Drug	
Myricetin	3.1 ± 0.2	3.6 ± 0.4	116.6 ± 12.9	ND	10
		29.0 ± 7.3			
Quercetin	3.0 ± 0.2	6.2 ± 0.3***	94.5 ± 7.7	75.9 ± 4.3*	6
Genistein	3.1 ± 0.4	3.8 ± 0.3*	112.0 ± 7.9	133.0 ± 11.5*	7
Isorhamnetin	2.4 ± 0.2	5.4 ± 0.4**	112.8 ± 11.4	89.1 ± 8.8*	5
Luteolin	2.5 ± 0.2	2.8 ± 0.3	115.8 ± 11.1	113.6 ± 15.3	5
Apigenin	2.9 ± 0.3	2.9 ± 0.3	100.2 ± 2.6	109.6 ± 8.2	6
Chrysin	3.2 ± 0.3	3.1 ± 0.2	97.0 ± 8.6	70.8 ± 3.2**	6
Kaempferol	2.5 ± 0.3	4.6 ± 0.3***	106.7 ± 4.6	101.9 ± 7.0	6
Tamarixetin	2.3 ± 0.1	4.6 ± 0.3***	97.1 ± 7.5	98.9 ± 10.3	9
Rutin	2.3 ± 0.2	2.4 ± 0.3	122.2 ± 11.2	117.4 ± 5.4	6
(±)-Taxifolin	2.7 ± 0.2	3.0 ± 0.3	117.2 ± 10.4	111.5 ± 5.5	6
3,6,4′-Trihydroxyflavone	2.7 ± 0.2	2.9 ± 0.3	116.7 ± 17.8	114.0 ± 15.7	5
5,7,2′-Trihydroxyflavone	3.2 ± 0.4	3.3 ± 0.3	104.6 ± 9.4	103.1 ± 4.7	5
Scutellarein	2.4 ± 0.2	2.7 ± 0.2	109.7 ± 8.7	114.0 ± 7.3	6
Naringin	2.8 ± 0.1	3.3 ± 0.3	122.4 ± 16.1	109.1 ± 14.1	4
5-Hydroxyflavone	3.0 ± 0.7	2.5 ± 0.4	92.0 ± 4.8	85.5 ± 1.4	4
3,4′-Dihydroxyflavone	2.7 ± 0.2	2.9 ± 0.3	100.8 ± 9.7	98.9 ± 10.4	5
Daidzein	2.9 ± 0.4	3.1 ± 0.2	92.2 ± 9.0	110.2 ± 12.9*	5
Morin	2.7 ± 0.2	3.2 ± 0.1*	127.9 ± 10.6	158.1 ± 16.8*	5
(±)-Naringenin	2.7 ± 0.2	2.9 ± 0.3	119.6 ± 13.5	98.5 ± 8.5*	5
Resokaempferol	2.6 ± 0.3	2.7 ± 0.4	114.4 ± 14.0	110.1 ± 9.6	5
Baicalein	2.6 ± 0.2	2.9 ± 0.4	102.9 ± 9.4	99.7 ± 5.7	6
3-Methyl galangin	2.6 ± 0.4	3.0 ± 0.5	114.9 ± 5.9	82.3 ± 10.0*	4
Galangin	2.2 ± 0.2	3.1 ± 0.5	99.3 ± 7.5	89.2 ± 5.1	5

*ND* not detectable

For myricetin, both τ_1act_ and τ_2act_ have been reported; data = mean ± SEM; * p < 0.05, ** p < 0.01, *** p < 0.001

**Fig. 3:**
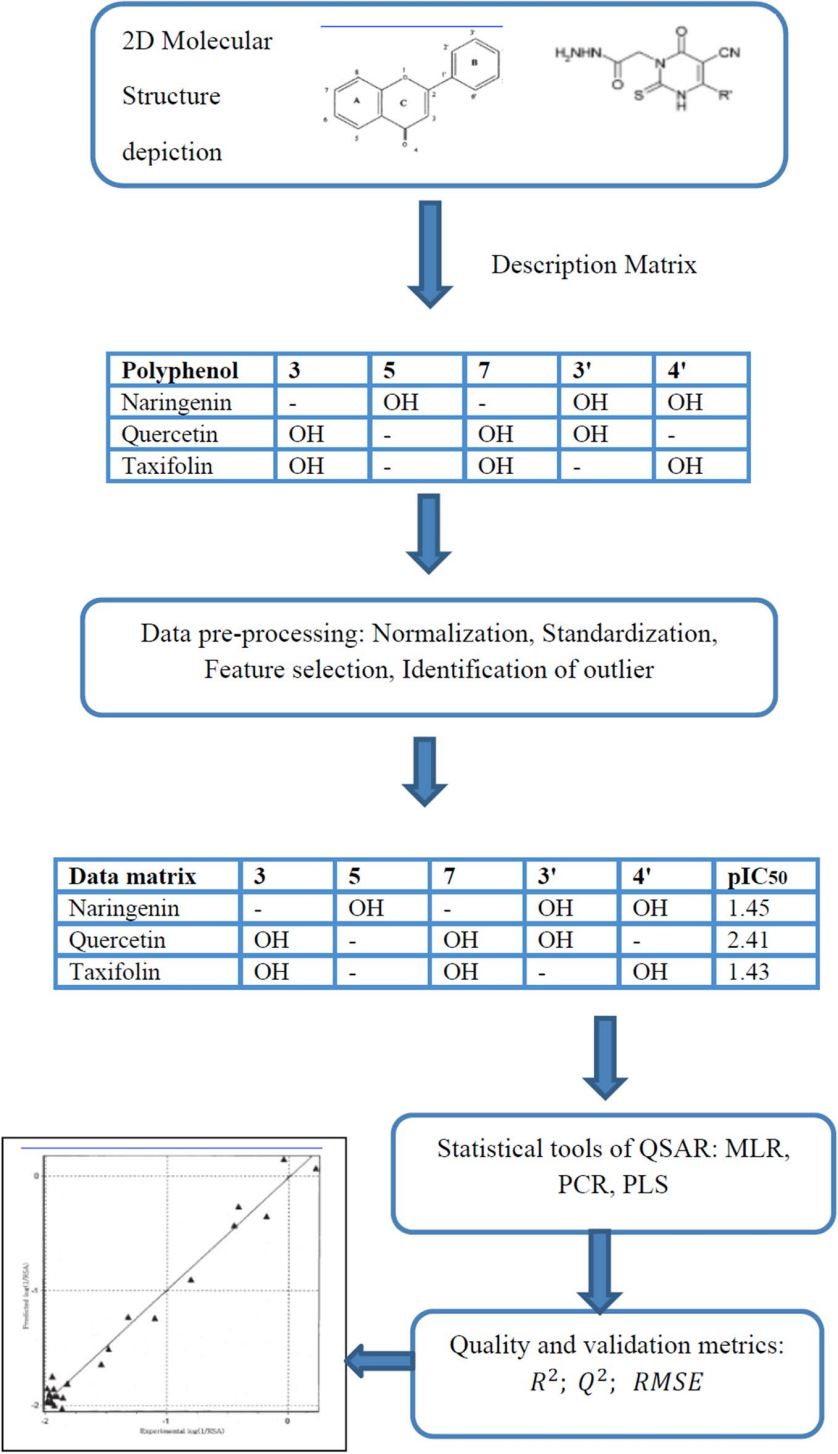
Flowcharts describing the ways of chemical structures used to calculate descriptors for QSAR model

The type of descriptors used and the extent to which they could encode the structural features of the molecules that were correlated to the response were critical determinants of the quality of the QSAR model. The ways of chemical structures used to calculate descriptors for QSAR model were illustrated in Fig. 3. The data set of flavonoids constituted a group of small polyphenol compounds which can both block and enhance Ca^2+^ current. Firstly, the half maximal activiting/inhibitory concentration (IC_50_) was regarded as the activatory/inhibitory activity values. Then, [IC_50_(μM)] that was referred as the activity data was transformed into the logarithmic scale pIC_50_, i.e. [− log IC_50_(μM)], that had been applied as the response variables to obtain the linear relationship in the QSAR equation. Secondly, the biochemical database of the study was randomly classified into two subsets that include 4 compounds of test-set and 20 compounds training-set (Table 3). Thirdly, the molecular descriptors were computed by the docking QSAR software for different types of theoretical descriptors for each flavonoid. Finally, two models, namely model A for CaV activation and model B for CaV inhibition, were generated by PCR after it screened for different combinations of descriptors by genetic algorithm.

**Table 3:** Flavonoids classified by its corresponding PubChem CID and training/test set

Flavonoids	PubChem CID	Training and test data set
5,7,2′-Trihydroxyflavone	Structure2D_CID_21611827	Training set
Naringin	Structure2D_CID_442428	Training set
(±)-Taxifolin	Structure2D_CID_471	Training set
Quercetin	Structure2D_CID_5280343	Training set
Apigenin	Structure2D_CID_5280443	Training set
Luteolin	Structure2D_CID_5280445	Training set
Rutin	Structure2D_CID_5280805	Training set
Kaempferol	Structure2D_CID_5280863	Training set
Genistein	Structure2D_CID_5280961	Training set
Baicalein	Structure2D_CID_5281605	Training set
Chrysin	Structure2D_CID_5281607	Training set
Resokaempferol	Structure2D_CID_5281611	Training set
Galangin	Structure2D_CID_5281616	Training set
Isorhamnetin	Structure2D_CID_5281654	Training set
Morin	Structure2D_CID_5281670	Training set
Myricetin	Structure2D_CID_5281672	Training set
Scutellarein	Structure2D_CID_5281697	Training set
Tamarixetin	Structure2D_CID_5281699	Training set
Daidzein	Structure2D_CID_5281708	Training set
3-Methyl galangin	Structure2D_CID_5281946	Training set
5-Hydroxyflavone	Structure2D_CID_68112	Test set
3,6,4′-Trihydroxyflavone	Structure2D_CID_688684	Test set
3,4′-Dihydroxyflavone	Structure2D_CID_688715	Test set
(±)-Naringenin	Structure2D_CID_932	Test set

### Validation on QSAR models

**Fig. 4:**
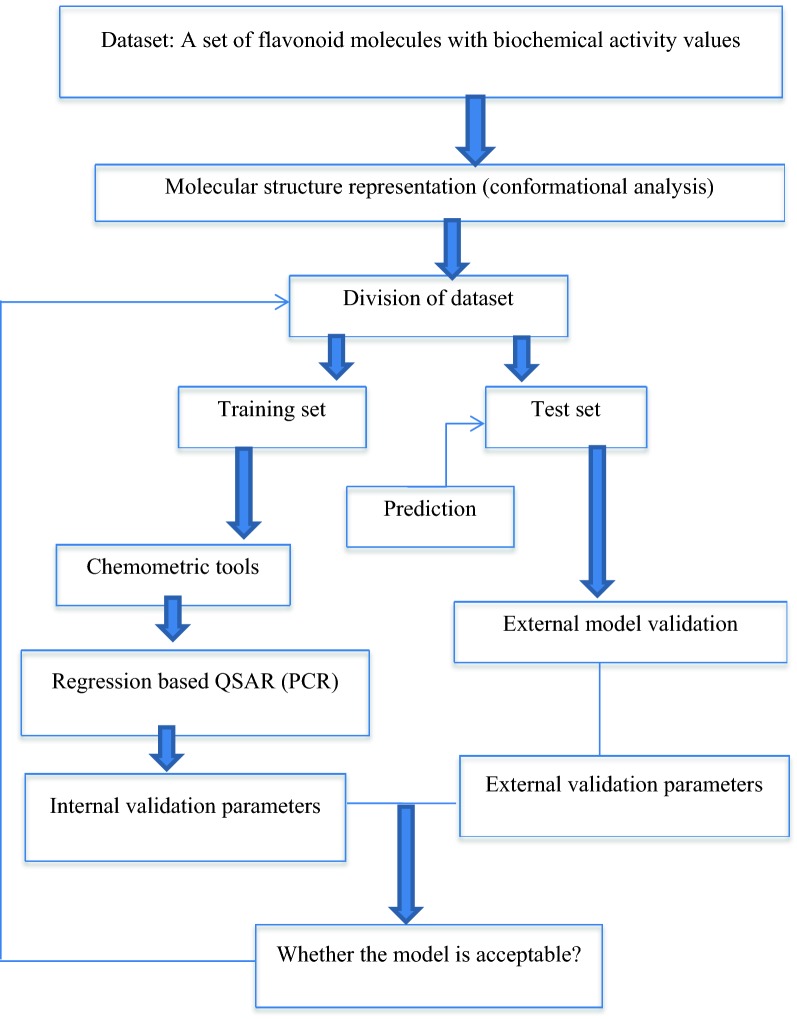
Steps of validation methods for the QSAR model

Although there are no confirmatory experiments performed to validate simulation results, these in silico data could be confirmed by other QSAR methods such as comparative molecular field analysis and support vector machine. Validation for QSAR model was done based on the flavonoids for detecting the precision of predictions. The leave-one-out cross validation technique was mainly involved in validating the sample (n = 24). For checking reliability of a QSAR model for prediction of the response property on the data, the original data set was classified into 6 subsets with each of size 4. The validation process was repeated by using 5 subsets as training set and the rest 4 as testing set. Training set was employed for model development while the ability of the model to predict response value of the flavonoids was done using the testing set. The developed models were subjected to statistical validation tests to establish its reliability. Steps of validation methods were indicated in Fig. 4. The following metrics for determination of QSAR quality, as well as internal and external validation were used:

- Metrics for determination of quality of QSAR model

Determination coefficient (r^2^):

$${\text{r}}^{2} = 1 - \frac{{\sum \left( {Y_{obs} - Y_{cal} } \right)^{2} }}{{\sum \left( {Y_{obs} - \overline{{Y_{obs} }} } \right)^{2} }} .$$

Adjusted r_a_^2^

$${\text{r}}_{a}^{2} = \frac{{\left( {{\text{N}} - 1} \right) \times r^{2} - p}}{N - 1 - p}.$$

Variance ratio (F)

$${\text{F}} = \frac{{\frac{{\sum (Y_{calc} - \bar{Y})^{2} }}{p} }}{{\frac{{\sum (Y_{obs} - Y_{calc} )^{2} }}{N - p - 1} }} .$$

Standard error of estimate (s)

$$S = \sqrt {\frac{{(Y_{obs} - Y_{cal} )^{2} }}{N - p - 1}} .$$

b. Validation metrics for QSAR model
  i. Internal validation

Leave-one-out (LOO) cross-validation

$$PRESS = \sum \left( {Y_{obs} - Y_{pred} } \right)^{2} ,$$

$${\text{SDEP}} = \sqrt {\frac{\text{PRESS}}{n}} ,$$

$${\text{q}}^{2} = 1 - \frac{{\sum \left( {Y_{{obs\left( {train} \right)}} - Y_{{pred\left( {train} \right)}} } \right)^{2} }}{{\sum \left( {Y_{{obs\left( {train} \right)}} - \bar{Y}_{training} } \right)^{2} }} = \frac{PRESS }{{\sum \left( {Y_{{obs\left( {train} \right)}} - \bar{Y}_{training} } \right)^{2} }} .$$

The $r_{m}^{2}$ metric for internal validation

$$\overline {{r_m}^2} = \frac{{\left( {{r_m}^2 + {{r_m^\prime }^2}} \right)}}{2},$$

$$\Delta {r_m}^2 = \left| {{r_m}^2 - {{r_m^\prime}^2}} \right|,$$

$$r_{m}^{2} = r^{2} \times \left( {1 - \sqrt {\left( {{\text{r}}^{2} - {\text{r}}_{0}^{2} } \right)} } \right),$$

$${{r_m^\prime}^2} = {r^2} \times \left( {1 - \sqrt {\left( {{r^2} - {{r_0^\prime }^2}} \right)} } \right),$$

$${\text{Scaled}}\;{Y_i} = \frac{{\left( {{r_m}^2 + {{r_m^\prime }^2}} \right)}}{2},$$

$${\text{Scaled}}\;Y_{i} = \frac{{Y_{i} - Y_{{\text{min} \left( {obs} \right)}} }}{{Y_{{\text{max} \left( {obs} \right)}} - Y_{{\text{min} \left( {obs} \right)}} }}.$$

ii. Metrics for external validation

Predictive r^2^_pred_

$${\text{r}}_{pred}^{2} = 1 - \frac{{\sum \left( {Y_{{obs\left( {test} \right)}} - Y_{{pred\left( {test} \right)}} } \right)^{2} }}{{\sum \left( {Y_{{obs\left( {test} \right)}} - \bar{Y}_{training} } \right)^{2} }} .$$

Root mean square error in prediction (RMSEP)

$${\text{RMSEP}} = \sqrt {\frac{{\sum \left( {y_{{obs\left( {test} \right)}} - y_{{pred\left( {test} \right)}} } \right)^{2} }}{{n_{ext} }}} .$$

iii. Molecular descriptor

“k2alpha” is descriptor indicating second order kappa alpha shape index (^2^kα or k2alpha):

$${\text{k}}2{\text{alpha}} = \frac{{\left( {A + \alpha - 1} \right)\left( {A + \alpha - 1} \right)^{2} }}{{\left( {P + \alpha } \right)^{2} }}.$$

“Id” and “IdwAverage” that are the type of information theory based descriptors on distance equality, whereas the total information content on the distance equality (Id):

$${\text{Id}} = \frac{{A\left( {A - 1} \right)}}{2}log_{2} \left( {\frac{{A\left( {A - 1} \right)}}{2}} \right) - \mathop \sum \limits_{g = 1}^{G} flog_{2} \left( f \right),$$

where f is the number of distances with equal g values in the triangular D submatrix. D is an A × A matrix that contains the graph distances between atoms. The graph distances are calculated as 1/(the number of bonds between atoms)^2^

The mean information content on the distance equality (IdwAverage):

$${\text{IdwAverage}} = - \mathop \sum \limits_{g = 1}^{G} \frac{2f}{{A\left( {A - 1} \right)}}log_{2} \left( {\frac{2f}{{A\left( {A - 1} \right)}}} \right).$$

## Results

As listed in Table 4, for Model A, the QSAR model not only had internal predictive ability ($q^{2}$ = 6.93%) and external predictive ability ($r^{2}_{pred}$ = 95.86%), but also could explain 31.82% of the total variance ($r^{2}$ =* 0.3182*) in the training database. The F-test = 3.9664 showed that the Model A was statistically significant with p-value < 0.001 for which it indicated the model had less than 0.1% probability of making an error. For Model B, although the QSAR model had only external predictive ability ($r^{2}_{pred}$ = 52.27%) and could only explains 8.45% of the total variance ($r^{2}$ = *0.0845*) in the training dataset, its internal predictive ability ($q^{2}$ = 11.48%) was relatively higher than that of Model A. Similarly, the F-test = *1.5682* also indicated the Model B was statistically significant with p < 0.001 which meant that the Model B’s likelihood of committing an error was less than 5%. Therefore, both Model A and B were justified for their internal and external predictive ability.

**Table 4:** The QSAR model with the corresponding parameters of estimates

*Model A (for activation activity)*		
pIC_50_ = − 0.0413 k2alpha − 0.0003 Id + 0.5530 IdwAverage − 3.1819		
*F*-*stat *= 3.9664 p-*value *< 0.001		
$r^{2}$ = 0.3182	$q^{2}$ = 0.0693	$r^{2}_{pred}$ = 0.9586
$r^{2} \left( {se} \right)$ = 0.0933	$q^{2} \left( {se} \right)$ = 0.1090	$r^{2}_{pred} \left( {se} \right)$ = 0.0204
*Model B (For inhibition activity)*		
pIC_50_ = 0.3241Most+vePotential + 0.0000MomInertiaY − 0.3600DeltaEpsilonC + 1.9116		
*F*-*stat *= 1.5682 p-*value *< 0.001		
$r^{2}$ = 0.0845	$q^{2}$ = 0.1148	$r^{2}_{pred}$ = 0.5227
$r^{2} \left( {se} \right)$ = 0.0804	$q^{2} \left( {se} \right)$ = 0.0887	$r^{2}_{pred} \left( {se} \right)$ = 0.0387

r^2^, determination coefficient; q^2^, internal predictive ability; r^2^_pred_, external predictive ability; se, standard error; pIC_50_, concentration of flavonoids in logarithm scale required for 50% activation/inhibition of CaV channel activity

**Fig. 5:**
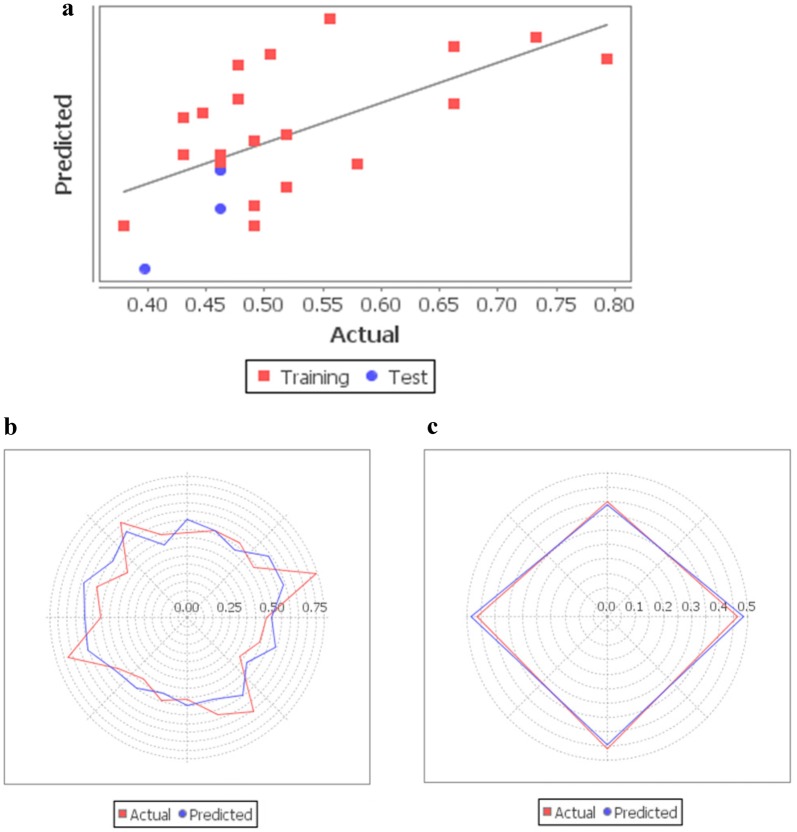
**a** Model A’s graph of goodness of fit indicating observed and predicted activity of polyphenols on CaV activation by QSAR equations along with the residuals, **b** Model A’s Radar plot depicting closeness between the actual and predicted activity of the flavonoid compounds of training set, **c** Model A’s Radar plot depicting closeness between the actual and predicted activity of the test set’s compounds

**Fig. 6:**
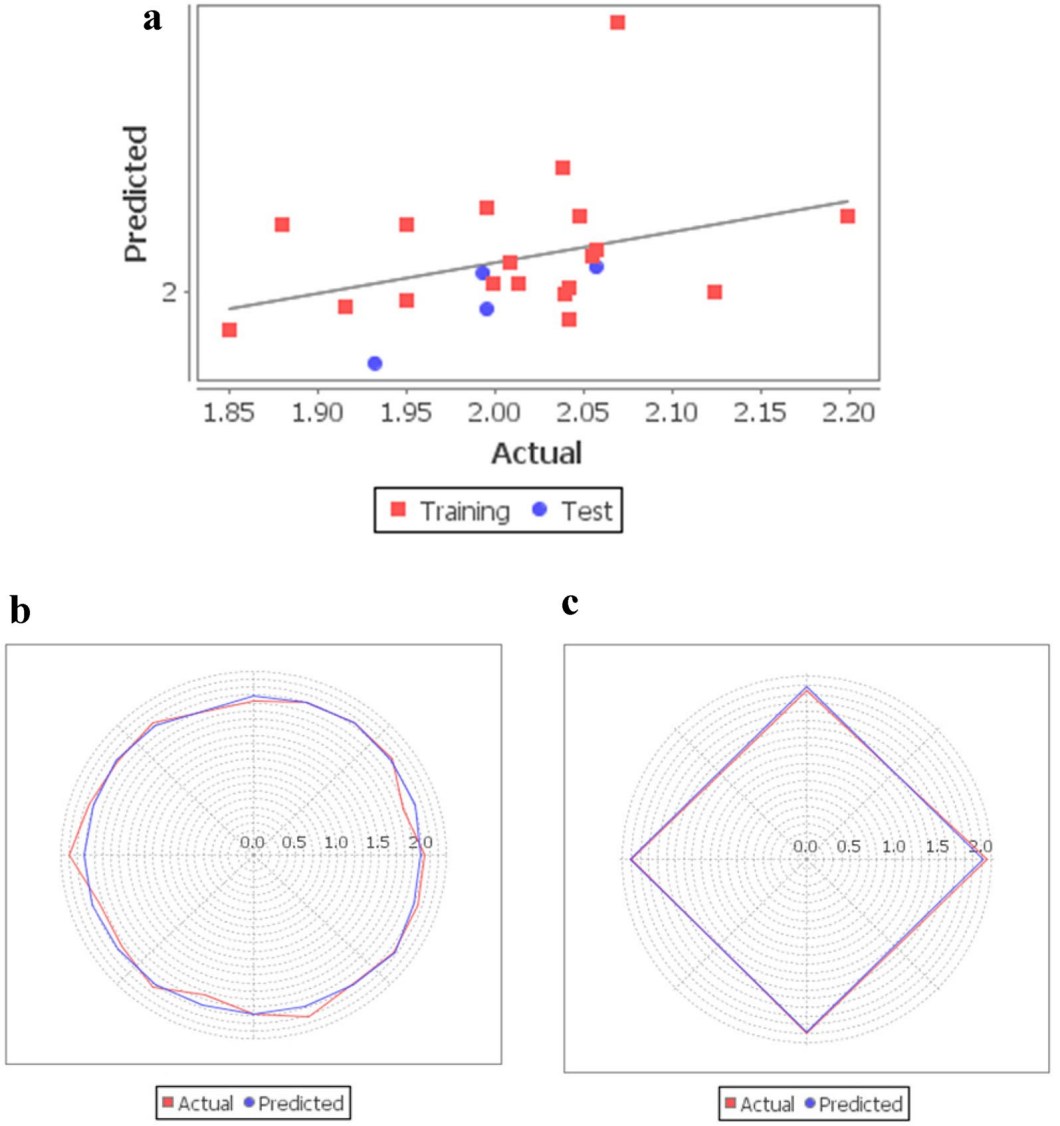
**a** Model B’s graph of goodness of fit indicating observed and predicted activity of polyphenols on CaV inhibition by QSAR equation along with the residuals, **b** Model B’s Radar plot depicting closeness between the actual and predicted activity of the flavonoid compounds of training set, **c** Model B’s Radar plot depicting closeness between the actual and predicted activity of test set’s compounds

Tables 5 and 6 indicate the observed values and the predicted values of the activation (Model A) and inhibition (Model B) activity of flavonoids on CaV channel, respectively. Figure 5a shows the goodness of fit graph of observed activity and predicted activity of the flavonoids activating on CaV channel. Moreover, it indicated how good the actual training dataset could be fitted by the predicted PCR equation. From the Radar plots as shown in Fig. 5b, c, the fitted PCR equation of the training data set could be predicted well by the test data set. Hence, the predictive ability of Model A could be confirmed. On the other hand, Fig. 6a indicates that the fitness plot of observed activity and predicted activity of the flavonoids inhibiting on CaV channel. In addition, it showed how well the actual training dataset could be fits by the predicted PCR equation. From Radar plots in Fig. 6b, c, it was also revealed that the fitted PCR equation of the training data set could be predicted well by the test data set. Therefore, the predictive ability of Model B could also be confirmed.

**Table 5:** Observed and predicted activity of the flavonoids on the CaV activation (Model A)

Flavonoids	Observed activity	Predicted activity	Residuals
5,7,2′-Trihydroxyflavone	0.519	0.518	− 0.001
Naringin	0.519	0.468	− 0.051
(±)-Taxifolin	0.477	0.584	0.107
Quercetin	0.792	0.590	− 0.202
Apigenin	0.462	0.490	0.028
Luteolin	0.447	0.539	0.092
Rutin	0.38	0.431	0.051
Kaempferol	0.663	0.547	− 0.116
Genistein	0.580	0.490	− 0.090
Baicalein	0.462	0.499	0.037
Chrysin	0.491	0.449	− 0.042
Resokaempferol	0.431	0.498	0.067
Galangin	0.491	0.512	0.021
Isorhamnetin	0.732	0.611	− 0.121
Morin	0.505	0.595	0.090
Myricetin	0.556	0.630	0.074
Scutellarein	0.431	0.534	0.103
Tamarixetin	0.663	0.602	− 0.061
Daidzein	0.491	0.431	− 0.060
3-Methyl galangin	0.477	0.552	0.075
5-Hydroxyflavone	0.398	0.389	− 0.009
3,6,4′-Trihydroxyflavone	0.462	0.484	0.022
3,4′-Dihydroxyflavone	0.462	0.447	− 0.015
(±)-Naringenin	0.462	0.484	0.022

**Table 6:** Observed and predicted activity of the flavonoids on the CaV inhibition (Model B)

Flavonoids	Observed activity	Predicted activity	Residuals
5,7,2′-Trihydroxyflavone	2.013	2.003	− 0.010
Naringin	2.038	2.042	0.004
(±)-Taxifolin	2.047	2.026	− 0.021
Quercetin	1.880	2.023	0.143
Apigenin	2.040	1.999	− 0.041
Luteolin	2.055	2.012	− 0.043
Rutin	2.070	2.091	0.021
Kaempferol	2.008	2.010	0.002
Genistein	2.124	2.000	− 0.124
Baicalein	1.999	2.003	0.004
Chrysin	1.850	1.987	0.137
Resokaempferol	2.042	2.002	− 0.040
Galangin	1.950	1.997	0.047
Isorhamnetin	1.950	2.023	0.073
Morin	2.199	2.026	− 0.173
Myricetin	2.057	2.015	− 0.042
Scutellarein	1.995	2.029	0.034
Tamarixetin	2.042	1.991	− 0.051
Daidzein	1.915	1.995	0.080
3-Methyl galangin	1.932	1.976	0.044
5-Hydroxyflavone	2.057	2.008	− 0.049
3,6,4′-Trihydroxyflavone	1.995	1.994	− 0.001
3,4′-Dihydroxyflavone	1.993	2.007	0.014

Table 7 shows the ranking the order of activation of flavonoids on the CaV channels such that the descending order of flavonoids activation on the CaV channels are: scutellarein > morin > daidzein > myricetin > apigenin > quercetin > (±)-taxifolin > 5,7,2′-trihydroxyflavone > genistein and so on.

**Table 7:** The order of the flavonoids ranked by relative contribution of individual descriptors using pIC_50_ in model A and the order of the compounds ranked by the proportion of increase in in vivo new bone formation

No.	Name	K2alpha	Id	IdwAverage	% Increase in new bone formation in vivo
1	Scutellarein	5.334	790.386	7.774	–
2	Morin	5.67	798.763	7.77	–
3	Daidzein	5.67	811.449	7.762	602% [10]
4	Myricetin	5.109	710.672	7.646	–
5	Apigenin	5.109	717.251	7.641	–
6	Quercetin	5.253	717.251	7.641	556% [11]
7	(±)-Taxifolin	12.063	3184.03	9.445	–
8	5,7,2′-Trihydroxyflavone	5.232	622.413	7.523	–
9	Genistein	4.885	645.406	7.502	520% [12]
10	Rutin	4.885	655.079	7.493	–
11	Kaempferol	12.328	3499.12	9.59	–
12	Tamarixetin	4.885	660.411	7.488	–
13	Naringin	4.502	560.369	7.37	490% [7]
14	Isorhamnetin	4.661	561.775	7.371	–
15	Chrysin	4.661	576.201	7.357	–
16	Galangin	4.661	578.975	7.357	–
17	Luteolin	4.661	586.848	7.347	–
18	Baicalein	4.661	587.476	7.347	–
19	Resokaempferol	4.438	508.035	7.208	–
20	3-Methylgalangin	4.438	528.263	7.187	–

## Discussion

The results indicated in Table 7 has shown the molecular structures, contribution graphs and parameters of selected descriptors of flavonoids as predicted in the CaV activation model with QSAR equations (model A) The results were surprisingly consistent with the order of the flavonoids ranked by the percentage of increase in new bone formation, i.e. daidzein (602%) > quercetin (556%) > genistein (520%) > naringin (490%) as reported in series of in vivo animal studies by Wong et al. [7, 10–12]. This partial external validation, despite not a full set of comparison, gives a good guarantee for the present predictive supervised machine-learning QSAR model that can precisely ranks and predicts the flavonoids effects on osteogenesis based on the existing biochemical information with CaV [19].

Activation and inhibition activities of flavonoids were investigated based on their ability to maintain the balance between activation and inactivation in the CaV by binding to the beta subunit receptor of CaV. In this case of activation, it was indicated that activating activity is mainly the outcome of electronic interactions between atomic charges within flavonoids and possible receptor-like structures in the CaV. In the case of inhibition, it was shown that the binding affinities of selected flavonoids to the CaV receptor are highly dependent on physicochemical properties involved in the interactions. As such, statistically the Model A presented the justified characteristics of molecular structure of flavonoid compounds that were required for activating CaV channel in which electrostatic fields were estimated using “k2alpha” that was descriptor signifying second alpha modified shape index, and “Id” and “IdwAverage” that were the type of information theory based descriptors. On the contrary, the Model B showed different structural features that were required for flavonoids to inhibit CaV channel. Its electrostatic fields were estimated using “Most+vePotential” that was descriptor indicating the highest value of positive electrostatic potential on the van der Waals surface area of the flavonoids, “MomInertiaY” that was steric descriptor signifying moment of inertia at Y-axis, and “DeltaEpsilonC” that was descriptor for electronegativity signifying differences between the frontier molecular orbital energies. Hence, from the six descriptors selected for the principal component regression model, one was related to the electronic (i.e. “Most+vePotential”) or two were related to the physicochemical (“MomInertiaY” and “DeltaEpsilonC”) properties of the whole molecules and three (“k2alpha”, “Id”, “IdwAverage”) described electronic properties of individual atoms. All these selected descriptors correspond to the analogous behavior in terms of tendency that was being also observed by the QSAR analysis on the CaV channel to be activated and inhibited by the flavonoids.

Furthermore, the QSAR model was chosen in accordance with the parameter estimates of $r^{2}$, $q^{2}$, $r^{2}_{pred}$, F-stat and p-value. Since the $r^{2}$ value = 0.3182 of Model A was higher than the Model B’s $r^{2}$ value = 0.0845, and the $q^{2}$ value = 0.0693 of Model A was relatively lower than the Model B’s $q^{2}$ value = 0.1148, and $r^{2}_{pred}$ = 0.9586 of Model A was higher than the Model B’s $r^{2}_{pred}$ = 0.5227, Model A had justified values for being selected to be the better QSAR model to support the argument that CaV channel was more likely to be activated rather than being inhibited by the flavonoids. From this point, the QSAR approach pursues its objective of understanding the biochemical effects of the flavonoids on the CaV channel and providing practical suggestions for screening optimal flavonoids have been demonstrated in the investigations of its activating and inhibitory activity on CaV channel. Moreover, analogous behavior in terms of selected descriptors tendency was also observed by the QSAR analysis on the CaV channel to be activated and inhibited by the flavonoids. Activation and inhibition activity of flavonoids was investigated based on their ability to maintain the balance between activation and inactivation in the CaV by binding to the beta subunit receptor of CaV. In this case of activation, it was indicated that activating activity is mainly the outcome of electronic interactions between atomic charges within flavonoids and possible receptor-like structures in the CaV. In the case of inhibition, it was shown that the binding affinities of selected flavonoids to the CaV receptor are highly dependent on physicochemical properties involved in the interactions.

Apparently, the electronic properties of the flavonoids were found to be significant after exploring the entire pool of the classical and electronic variables for screening a QSAR model which has thousands of parameters available from experiment and in silico calculations that could potentially serve as independent variables (descriptors) in statistical analysis. However, it has also been known from fitting this QSAR study that utilization of an excessive number of descriptors leads to over-fitting of QSAR models and/or increases the risk of chance correlations. Despite the existence of rules for building successful and meaningful QSAR models, the increasing complexity of biochemical mechanisms the flavonoids on the CaV creates the need for considering a large variety of variables that makes the knowledge-based approach to the identification of the most significant descriptors for this particular case of investigating flavonoids’ biochemical activities on the CaV channel extremely difficult. Therefore, this is the main reason to apply PCR to perform reduction of data by generating linear combinations of molecular descriptors [27]. The PCR method identifies correlated variables, groups them into linear combinations, and generates uncorrelated orthogonal variables that are uncorrelated and called principal components. The process of data transformation is given by ${\text{X}} = TP^{T}$, where X represents the initial data matrix, T is a score matrix that defines the position of data points in a new coordinate system and P is a loadings matrix. The loadings indicate how much each original descriptor contributes to the corresponding PC. Scores and loadings allow the data points to be mapped into the new vector space defined by PCs [28].

The correlation between independent and dependent variables could statistically be determined to fit a PCR line to the data so as to obtain a best-fit equation. Then, the goodness of fit for a PCR equation was estimated by referring to its standard deviation and correlational coefficient in which the level of statistical significance of the PCR equation was represented by the F-statistics with its corresponding p-value. By applying PCR analysis, enough PCs could be used to do variable selection by choosing p-value in order to maximize adjusted r^2^. We notice that the limitation of this study could be only ascribable to 24 compounds, and the predictive power could be less using this small dataset. Indeed, various QSAR studies [29–31] have used 10–25 compounds to generate the predictive model that seems to be quite successful. On the other hand, regarding to the results, although it is generally recommended that *r*^2^ should be > 0.7, and *q*^2^ should be > 0.5, these are not stringent guidelines. The predictive power of QSAR should not solely rely on *r*^2^ and *q*^2^ [32]. In particular, we have also cross-compared with the in vivo animal studies (Table 7) for daidzein, quercetin, genistein and naringin [7, 10–12]. If necessary, further time-consuming in vivo studies could be done in order to completely validate the model.

In this study the combination of electronic and physicochemical descriptors helped to identify molecular shape, hydrophobicity and electronic properties as three major factors responsible for these types of activation and inhibition activity of flavonoids on the CaV. The use of QSAR in screening the bioactive flavonoids for tissue engineering applications is relatively new. The success of this QSAR modeling in the accurate determination of electronic properties of biochemically significant flavonoids may initiate QSAR studies in tissue engineering that focus specifically on the exploration of bioactive growth factors for cells. QSAR provides an invaluable tool for calculating quantum-chemical descriptors that demonstrate high potential in generating predictive QSAR models without the addition of a large number of descriptors for various groups of growth factors [33, 34]. Since osteogenesis is a very dynamic process, other factors that are related to CaV channel such as runx2 activation, ALP secretion, osteocalcin level, angiogenesis and mineralization can also be incorporated if appropriate in the future study. Nevertheless, cautious should be taken into account for QSAR studies because it is only an approximating method. When many physicochemical properties are involved, it is not always possible to vary one property without affecting another. Moreover, it does not provide an in-depth insight on the mechanism of biological action of flavonoids. Also, there may be some risk of inaccurate predictions of biological activity of this type of flavonoid compounds.

Through the QSAR study, we have established a predictive molecular modeling method that allows one to estimate the properties of flavonoids as bioactive compounds at a much lower cost and environmentally-friendly than that of actual laboratory screening. Since both the model’s predictive ability and the scientific insights into biochemical activity in the CaV depend on the descriptors selected in the modeling process, this study has indicated that the use of quantum-chemical descriptors under supervised machine-learning has an obvious advantage over other experimentally measured properties. Since they are reproducible in the framework of the chosen approximation, they allow meaningful interpretation of QSAR models in terms of the biochemical mechanism of flavonoids as activator of the CaV. Thus, it can offer a clear guidance for molecule optimization or design of flavonoids as a growth factor for osteogenesis.

## Conclusions

This predictive QSAR study confirmed and validated the biochemical activity of the flavonoids in the CaV, such that flavonoids can activate CaV in osteogenesis. Scutellarein was predicted to rank the highest among the screened flavonoids.

## Data Availability

The datasets during and/or analysed during the current study available from the corresponding author on reasonable request.

## Abbreviations

CaV: Voltage-gated calcium
LPS: Lipopolysaccharide
QSAR: Quantitative structure–activity relationship
TNF-α: Tumor necrosis factor alpha
PLS: Partial least squares
MLR: Multiple linear regression
GA: Genetic algorithm
PCs: Principle components
PCR: Principle components regression
IC50: Half maximal inhibitory concentration
